# The time elapsed between assessments of blood metabolome and live weight affects associations between the abundance of metabolites and growth rate in beef cattle

**DOI:** 10.1007/s11306-023-02015-9

**Published:** 2023-05-15

**Authors:** José Augusto Imaz, S. C. Garcia, L. A. González

**Affiliations:** 1 Sydney Institute of Agriculture, School of Life and Environmental Sciences, Faculty of Science, The University of Sydney, Sydney, NSW 2570 Australia; 2 Dairy Research Foundation, Sydney, Australia; 3 Department of Regional NSW, Primary Industries, Menangle, Sydney, NSW Australia

**Keywords:** Live weight, Interval, Growth rate, Metabolome, Associations

## Abstract

**Introduction:**

This study aimed to assess the associations between the relative abundance (RA) of blood metabolites and growth rate (i.e., live weight change, LWC) calculated using different intervals of time between live weight (LW) measurements from the metabolome assessment.

**Methods:**

Grazing beef cattle were raised for 56 days and blood samples from each animal were taken on day 57. Live weight was continuously measured using an automatic in-paddock weighing scale. The RA of plasma metabolites were determined using proton nuclear magnetic resonance (NMR). Live weight data were filtered for outliers and one LW record was selected every 1, 7, 14, 21, 28, 35, 42, 49 and 56 days before the metabolome assessment (LWC1 to LWC56, respectively). Live weight change was then re-calculated for each interval between LW data selected.

**Results:**

Associations between LWC calculations and the RA of metabolites were greatly affected by the interval of time between LW data selected. Thus, the number of significant associations decreased from 9 for LWC1 to 5 for LWC35 whereas no significant associations were found for LWC56 (P > 0.05). There were 7 metabolites negatively associated with LWC1 including leucine, 2-hydroxybutyrate, valine, creatinine, creatine, phenylalanine and methylhistidine; however, correlations were positive for 2 lipids. The strength of the correlation coefficients decreased as the length of the interval between LW measures increased although this reduction was greater for some metabolites such as leucine compared to others such as lipids. Our findings suggest that the time frame in which a particular response variable, such as LWC, is measured and metabolomic samples are taken could largely impact associations and thus conclusions drawn.

**Conclusions:**

Depending on the variable to be explored, rapid changes in cattle metabolome may not be reflected in correlations if they are not assessed close in time. Our findings suggest that LWC should be measured for a period shorter than 28 days before the metabolome assessment as the number of significant associations decreases when LWC is measured for longer periods.

## Introduction

Assessing live weight (LW) in cattle over time is key to calculate growth rate (i.e., live weight change, LWC) and to study its associations with data obtained as a result of analysing biological tissues such as blood, saliva or faeces (Fontanesi, 2016). For instance, metabolomics is a discipline that identifies and quantifies a large number of metabolites in biological samples (Fontanesi, 2016). Cattle metabolome has been used to understand the metabolic processes linked to growth rate; however, some studies sampled animals at a single point in time at the beginning (Connolly et al., 2019) or the end of long experimental periods (Ogunade & McCoun, 2020). Therefore, that sample may represent the metabolome at that particular point in time only whereas growth rate was calculated over periods ranging from a few weeks to several months. Thus, the lack of frequent LW measurements to calculate LWC closer in time to the metabolome assessment may affect the number and strength of associations with the animals’ metabolome that studies could reveal. Nowadays, in-paddock weighing scales enable near real-time monitoring of LW in grazing cattle, remotely and automatically ((Imaz et al., 2020a). As a result, daily LW measurements obtained can be used to explore associations between cattle metabolome and LWC calculated using different intervals between LW records. This information could aid to better understand metabolic processes involved in animal growth and development by identifying key metabolites linked with long, medium and short term growth rates.

The present study aimed to assess the associations between the relative abundance (RA) of blood metabolites and LWC calculated at increasing lengths of time between LW measurements in grazing beef cattle. We hypothesised that LWC calculations at shorter intervals of time between LW data affect the number and strength of associations between animals’ metabolome and LWC.

## Methods

The study had animal ethics approval from The University of Sydney Animal Ethics Committee: Protocol no. 2017/1162. The study was undertaken following the Australian code for the care and use of animals for scientific purposes 8th Edition 2013.

### Study design

Twenty-two heifers and thirty steers (initial age 219 ± 50 days; initial LW 186 ± 35.1 kg/hd) grazed pastures and annual crops for 56 days from 14 June to 09 August 2017 at John Pye Farm (The University of Sydney, NSW). Feed types consumed during the study were: (a) Autumn temperate pastures grazed from day 0 to 28 (7.37% crude protein); (b) Oat crops grazed from day 29 to 56 (10.83% crude protein). A two-section yard centrally located to the paddocks (15 m x 25 m) was built enclosing the only water point which contained an in-paddock weighing scale with an auto drafter gate at the entry of the yard to measure LW every time the animals walk through the weighing platform (Precision Pastoral Ltd, Alice Spring, Northern Territory, Australia). Twenty-seven animals were randomly assigned and automatically drafted to one section of the yard containing an electronic feeder, in which a single molasses-lick block (MLB) was offered as a free choice (Smartfeed developed by C-lock Inc., Rapid City, South Dakota, United States of America). Further description of the setup of technologies and experimental details can be found in Imaz et al. (2019, 2020b).

### Blood sample obtention and preparation for metabolome profiling

**Fig. 1:**
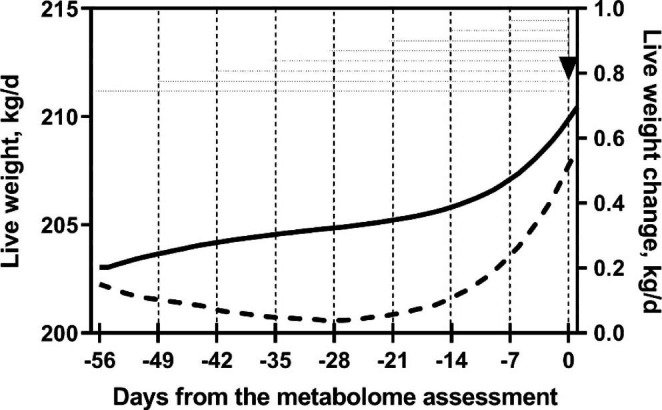
Scheme of the experiment including live weight (LW, left axis, continuous line) and live weight change (LWC, right axis, dashed line) of grazing cattle measured using automatic weighing for 56 days. The arrow indicates the blood metabolome assessment and horizontal dashed lines represent intervals of time between LW measures

A blood sample from each animal was obtained via puncture of the coccygeal vein using evacuated tubes (containing EDTA) on the morning of 10 August (day 0, Fig. 1). Blood samples were refrigerated at 4 ºC for approximately 20 min, then centrifuged (2000 × g for 30 min). Plasma was harvested and stored at -80 ºC.

The RA of metabolites was determined by proton nuclear magnetic resonance (1 H-NMR). Sample preparation and analysis were performed following the published methodology by Dona et al. (2014) at the facilities of Sydney Analytical (The University of Sydney, Australia).

Data were analysed using Matlab 7.0 Software (Matworks, Natick, MA). The spectra were aligned and normalised, automatically phased, baseline corrected and referenced to the α-C1H-Glucose doublet (5.233 ppm). The residual water (2.42–3.14 ppm) was truncated from the dataset to reduce analytical variability. The normalised spectra were then subjected to Standard Recoupling of Variables to obtain clusters (or components or features).

The cluster value for each sample is the area under the curve for each cluster (component or peak). These values are used as relative concentrations and were multiplied by 10^6^ to reduce the number of decimal places before analysis. Also, Chenomx®, existing literature and the Livestock Metabolome Database (Goldansaz et al., 2017; Nicholson et al., 1995; Weljie et al., 2006) were used for metabolites’ identification from raw data. Finally, the RA of the metabolites identified was calculated by adding up the relative concentration of peaks belonging to the same metabolite. Further description of animal management and blood samplings can be found in Imaz et al. (2022).

### Data processing and statistical analysis

Data recorded by the automatic scale were filtered for outliers and then smoothed using penalised b-splines (González et al., 2014; (Imaz et al., 2020a). Daily LWC (kg/d) was calculated from the smoothed data as the first derivative of the predicted LW curve. Then, the resulting LW and LWC data were averaged by date for each animal if more than one measurement per animal and day existed. Interpolation between days with LW measurements was done so all animals had data every day throughout the experimental period. This dataset was then used to create subsets of data selecting intervals of LW data for each animal ranging from 1 to 56 days between two consecutive LW measurements. Thus, one LW record was selected every 1, 7, 14, 21, 28, 35, 42, 49 and 56 days before blood samples were obtained. Live weight change was then re-calculated for each interval of time between LW data utilised resulting in LWC calculated for 1, 7, 14, 21, 28, 35, 42, 49 and 56 days before the metabolome assessment (Fig. 1; LWC1, LWC7, LWC14, LWC21, LWC28, LWC35, LWC42, LWC49 and LWC56, respectively).

The RA of glutamine, formate, glycine, dimethyl sulfone and lactate were transformed to log10 before analysis to normalise their distribution. No significant effects of sex and MLB supplementation on LWC calculations were detected using linear models (P > 0.05). Thus, Pearson’s correlation analysis was done between the RA of metabolites and each LWC calculation. Significant statistical differences were declared at P ≤ 0.05. All statistical analyses were done using SAS 9.4 (SAS Institute Inc., Cary, New Jersey, USA).

## Results

Twenty-seven blood metabolites were identified and their relative abundance is presented in Table 1 (mean ± SD). Additionally, LWC calculations greatly varied using different intervals of time between LW measurements, being nearly 4 times greater for LWC1 than LWC56 (Table 2; P < 0.05). Large impacts on LWC calculations can be observed amongst individual animals. For instance, the difference between the maximum and minimum for LWC1 was 1.120 kg whereas the difference was 0.372 kg for LWC56.

**Table 1:** Descriptive statistics of the relative abundance of blood metabolites

Metabolite	Mean	SD	Minimum	Maximum
Glucose	3945.6	426.29	2993.7	4598.2
Valine	606.6	56.77	501.4	759.1
3-hydroxybutyrate	356.1	68.89	248.9	566.4
Glutamine	271.0	31.64	196.5	339.8
Acetyl groups	256.7	21.78	214.8	318.4
Glycine	223.7	43.76	144.0	327.34
Acetate	171.8	39.34	86.5	258.9
Citrate	170.3	28.52	89.4	235.1
VLD lipids^*1^	160.3	44.53	80.1	289.7
2-hydroxybutyrate	153.3	15.60	122.7	189.8
Leucine	129.3	14.18	106.1	163.2
Creatine	117.8	32.56	62.4	230.5
LD lipids^*2^	117.2	19.62	75.2	174.9
Lactate	111.1	38.66	65.6	226.9
Alanine	95.3	6.96	80.4	111.2
Isoleucine	83.4	10.59	62.1	113.8
Threonine	82.3	12.57	53.8	106.7
Unsaturated lipid	81.1	35.98	23.3	159.2
Choline	73.0	13.30	45.8	113.6
Pyruvate	61.1	7.60	49.3	81.7
Phenylalanine	39.7	6.21	30.6	60.7
Dimethylsulfone	38.6	10.17	26.7	82.0
Creatinine	38.5	5.45	28.5	51.9
Tyrosine	28.6	4.71	20.1	46.4
Methylhistidine	26.5	5.82	17.1	41.5
Mannose	15.4	3.55	10.7	30.7
Formate	9.6	2.19	6.3	16.4

^*1^ Very low-density lipids.

^*2^ Low-density lipids.

**Table 2:** Descriptive statistics of live weight change (LWC) calculated using different intervals of time between live weight (LW) measurements in grazing cattle ranging from 1 to 56 days from the blood metabolome assessment. Means between rows without a common superscript differ

Item	Mean	SE	Minimum	Maximum
LWC1	0.507 a	0.025	-0.023	1.097
LWC7	0.403 b	0.025	-0.167	0.932
LWC14	0.290 c	0.025	-0.215	0.779
LWC21	0.193 d	0.025	-0.143	0.519
LWC28	0.167 de	0.025	-0.142	0.484
LWC35	0.144 de	0.025	-0.100	0.423
LWC42	0.128 e	0.025	-0.091	0.348
LWC49	0.119 e	0.025	-0.074	0.324
LWC56	0.114 e	0.025	-0.061	0.311

Associations between LWC calculations and the RA of metabolites changed according to the interval of time between LW data utilised (Table 3). The P-values of most metabolites significantly associated with LWC1 increased as LWC calculations were done using longer intervals between LW data selected (i.e., from LWC1 to LWC56; Table 3, P < 0.05).

**Table 3:** P-values obtained from a Pearson’s correlation analysis between the relative abundance of blood metabolites and live weight change (LWC) calculated using live weight (LW) data at different intervals of time in grazing cattle

Metabolite	LWC1	LWC7	LWC14	LWC21	LWC28	LWC35	LWC42	LWC49	LWC56
Leucine	< 0.001	< 0.001	0.0003	0.0003	0.0036	0.0438	0.2201	0.4097	0.4461
2-hydroxybutyrate	0.0007	0.0006	0.0019	0.0019	0.0114	0.0777	0.3190	0.5844	0.6999
Valine	0.0014	0.0005	0.0010	0.0010	0.0038	0.0251	0.1187	0.2429	0.2975
Creatinine	0.0020	0.0015	0.0024	0.0024	0.0111	0.0517	0.1608	0.2737	0.3552
Creatine	0.0023	0.0063	0.0173	0.0173	0.0573	0.1877	0.4351	0.6647	0.7255
Unsaturated lipids	0.0217	0.0095	0.0102	0.0102	0.0164	0.0376	0.0967	0.1653	0.2280
Phenylalanine	0.0281	0.0416	0.0491	0.0491	0.0834	0.1900	0.4152	0.6476	0.8542
Methylhistidine	0.0312	0.0206	0.0248	0.0248	0.0566	0.1319	0.2637	0.3679	0.4450
VLD lipids^*1^	0.0477	0.0205	0.0169	0.0169	0.0237	0.0413	0.0779	0.1135	0.1579
Acetyl groups	0.0643	0.0801	0.1654	0.1654	0.4108	0.8561	0.7069	0.5530	0.6224
Pyruvate	0.0747	0.0442	0.0362	0.0362	0.0398	0.0632	0.1012	0.1392	0.1612
Formate	0.0863	0.1562	0.1702	0.1702	0.2126	0.3339	0.5028	0.6108	0.6263
Mannose	0.0921	0.0760	0.0861	0.0861	0.0847	0.1164	0.1895	0.2753	0.3115
Choline	0.1101	0.0460	0.0332	0.0332	0.0304	0.0406	0.0701	0.1124	0.1543
Isoleucine	0.1325	0.0738	0.0775	0.0775	0.1034	0.1530	0.2084	0.2182	0.2055
Citrate	0.1536	0.1825	0.1520	0.1520	0.1198	0.0807	0.0514	0.0404	0.0607
LD lipids^*2^	0.1662	0.1090	0.1043	0.1043	0.1166	0.1175	0.1076	0.0807	0.0732
Glucose	0.2405	0.3852	0.5189	0.5189	0.8001	0.7662	0.3900	0.1967	0.1269
Lactate	0.2951	0.1615	0.1239	0.1239	0.1366	0.1898	0.2698	0.3719	0.4784
3-hydroxybutyrate	0.3041	0.2225	0.2849	0.2849	0.4211	0.6002	0.7929	0.8893	0.9458
Glycine	0.3355	0.2331	0.1872	0.1872	0.1879	0.2188	0.2989	0.3879	0.5493
Threonine	0.3540	0.2937	0.2459	0.2459	0.3724	0.6686	0.8441	0.4711	0.2636
Dimethylsulfone	0.4916	0.5965	0.7611	0.7611	0.9180	0.9530	0.8536	0.8076	0.7741
Acetate	0.6278	0.6174	0.7135	0.7135	0.8319	0.8800	0.8810	0.8463	0.8492
Tyrosine	0.7399	0.9607	0.9463	0.9463	0.9307	0.9004	0.8732	0.8663	0.9438
Alanine	0.8732	0.6710	0.5785	0.5785	0.5709	0.6009	0.6551	0.6242	0.5653
Glutamine	0.9289	0.9227	0.9875	0.9875	0.9511	0.8447	0.7133	0.5927	0.5342
Number P < 0.05	9	11	11	11	8	5	0	1	0
Number P < 0.10	4	3	2	2	4	4	4	1	2

^*1^ Very low-density lipids.

^*2^ Low-density lipids.

^*3^ Number P < 0.05 and P < 0.10 at the bottom of the table indicate the number of metabolites significantly affected by LWC calculations at the level of 0.05 and 0.10, respectively.

There were 9 metabolites significantly associated with LWC1 (Table 3, P < 0.05; leucine, 2-hydroxybutyrate, valine, creatinine, creatine, unsaturated lipids, phenylalanine, methylhistidine and very-low-density -VLD- lipids). However, the number of significant associations decreased to 5 for LWC35 and there were no significant associations for LWC56. Also, acetyl groups, pyruvate, formate and mannose tended to be associated with LWC1 (P < 0.10, Table 2) whereas no significant associations with any LWC calculation were observed for the rest of the metabolites. Interestingly, some metabolites, such as low density -LD- lipids and citrate, have shown an increase in the level of significance as the interval of time between LW measurements increased.

Table 4 shows that Pearson’s correlation coefficients of those metabolites significatively associated with LWC1 decreased as LWC calculations were done using longer intervals between LW data. For instance, the correlation coefficient for Leucine was five times greater for LWC1 in comparison LWC56.

**Table 4:** Correlation coefficients obtained from a Pearson’s correlation analysis between the relative abundance of blood metabolites and live weight change (LWC) calculated using live weight (LW) data at different intervals of time in grazing cattle

Metabolite	LWC1	LWC7	LWC14	LWC21	LWC28	LWC35	LWC42	LWC49	LWC56
Leucine	-0.5365	-0.5544	-0.5015	-0.5015	-0.4118	-0.2923	-0.1803	-0.1218	-0.1126
2-hydroxybutyrate	-0.4737	-0.4801	-0.4362	-0.4362	-0.3623	-0.2571	-0.1469	-0.0810	-0.0571
Valine	-0.4486	-0.4815	-0.4618	-0.4618	-0.4096	-0.3230	-0.2282	-0.1718	-0.1535
Creatinine	-0.4353	-0.4463	-0.4282	-0.4282	-0.3633	-0.2825	-0.2057	-0.1612	-0.1364
Creatine	-0.4292	-0.3890	-0.3422	-0.3422	-0.2763	-0.1935	-0.1153	-0.0642	-0.0520
Unsaturated lipids	0.3306	0.3707	0.3675	0.3675	0.3446	0.3010	0.2426	0.2035	0.1773
Phenylalanine	-0.3171	-0.2953	-0.2856	-0.2856	-0.2525	-0.1925	-0.1203	-0.0677	-0.0273
Methylhistidine	-0.3115	-0.3334	-0.3237	-0.3237	-0.2770	-0.2206	-0.1646	-0.1329	-0.1129
VLD lipids^*1^	0.2873	0.3337	0.3432	0.3432	0.3262	0.2957	0.2569	0.2314	0.2071
Acetyl groups	-0.2692	-0.2551	-0.2035	-0.2035	-0.1215	-0.0269	0.0557	0.0878	0.0729
Pyruvate	-0.2597	-0.2918	-0.3032	-0.3032	-0.2978	-0.2703	-0.2395	-0.2166	-0.2055
Formate	-0.2502	-0.2079	-0.2013	-0.2013	-0.1832	-0.1425	-0.0991	-0.0753	-0.0721
Mannose	-0.2458	-0.2586	-0.2504	-0.2504	-0.2515	-0.2297	-0.1927	-0.1607	-0.1492
Choline	0.2336	0.2895	0.3081	0.3081	0.3128	0.2967	0.2638	0.2321	0.2088
Isoleucine	-0.2202	-0.2605	-0.2573	-0.2573	-0.2379	-0.2095	-0.1849	-0.1810	-0.1860
Citrate	-0.2092	-0.1957	-0.2100	-0.2100	-0.2276	-0.2547	-0.2828	-0.2970	-0.2728
LD lipids^*2^	0.2031	0.2343	0.2374	0.2374	0.2295	0.2289	0.2352	0.2546	0.2609
Glucose	0.1727	0.1282	0.0954	0.0954	0.0375	-0.0441	-0.1269	-0.1897	-0.2234
Lactate	-0.1543	-0.2054	-0.2251	-0.2251	-0.2180	-0.1926	-0.1625	-0.1318	-0.1048
3-hydroxybutyrate	-0.1515	-0.1794	-0.1575	-0.1575	-0.1188	-0.0776	-0.0389	-0.0206	-0.0101
Glycine	-0.1421	-0.1754	-0.1937	-0.1937	-0.1934	-0.1808	-0.1531	-0.1275	-0.0886
Threonine	-0.1368	-0.1547	-0.1708	-0.1708	-0.1317	-0.0634	0.0291	0.1065	0.1646
Dimethylsulfone	-0.1017	-0.0784	-0.0450	-0.0450	-0.0153	0.0087	0.0274	0.0361	0.0425
Acetate	-0.0718	-0.0740	-0.0544	-0.0544	-0.0315	-0.0224	-0.0222	-0.0287	-0.0282
Tyrosine	-0.0492	-0.0073	-0.0100	-0.0100	-0.0129	-0.0186	-0.0237	-0.0250	-0.0104
Alanine	-0.0237	-0.0629	-0.0822	-0.0822	-0.0839	-0.0774	-0.0662	-0.0725	-0.0851
Glutamine	0.0132	0.0144	0.0023	0.0023	-0.0091	-0.0290	-0.0544	-0.0792	-0.0920

^*1^ Very-low-density lipids.

^*2^ Low-density lipids.

## Discussion

The present study aimed to determine the impacts of LWC calculated using different intervals of time between LW measurements on associations with the RA of blood metabolites in grazing cattle. We explored such associations using LW data collected remotely which enabled LWC calculations at different intervals from a single point in time when cattle metabolome was assessed. Our results indicate a large effect of LWC calculations on the number and strength of significant associations. For instance, more than 50% of the significant correlations were lost at LWC35 in comparison with LWC1 whereas no significant correlations were detected at LWC56. Overall, the level of significance and strength of the correlation coefficients between LWC and the RA of individual metabolites was severely affected when LWC was calculated for periods longer than 4 weeks before the metabolome assessment. Studies reported such impacts of using LW data at different intervals for LWC calculations over time in different cattle categories ((Imaz et al., 2020a). However, we believe this is the first study investigating its impacts on the number and strength of correlations with metabolites. Other studies have investigated associations between cattle metabolome and live weight (Sikka et al., 1994), growth rate and feed efficiency (Ogunade & McCoun, 2020) and reproductive performance amongst cattle breeds (Gómez et al., 2020). However, animal assessments to be correlated with the metabolites’ abundance in these studies were mostly recorded at a single point in time during the whole trial period or the beginning and the end of the study and then values were averaged. Therefore, these studies are not comparable with the present study that measured LW frequently which enabled different LWC calculations to explore its implications. The presence and changes in the abundance of certain metabolites at a particular point in time are products of different metabolic pathways driving growth physiology in cattle (Fontanesi, 2016). Thus, from the purview of the present study, the existence of weak or the lack of associations between the abundance of blood metabolites and LWC could indicate that the metabolic profile of animals may be more closely associated with LWC measured close in time from the metabolic assessment. It is speculated that such effects are due to the rapid changes in the metabolome and thus, LWC should also be measured with a short timescale from when samples were taken. Also, it is important to highlight that the present study focused on the associations between the RA of metabolites and LWC and our findings could not be extrapolated to other variables, such as fat accretion or diseases, which may involve other physiological pathways and show different associations with animals’ metabolome.

Direct comparisons of our results with previous studies are difficult due to the uniqueness of the present study. However, our findings suggest that the existence and interpretation of associations between cattle growth and their metabolome could radically change based on the time distance between the metabolome assessment and LW measures. In this regard, Connolly et al. (2019) reported that the abundance of three metabolites (3-hydroxybutyrate, creatinine and aspartate) was correlated with the growth rate of feedlot cattle. However, Connolly et al. (2019) calculated animals’ growth rates by measuring LW at the beginning and the end of a 450-day feeding period. Another study (Ogunade & McCoun, 2020), fed beef weaners for 42 days and explored associations between their growth rate and metabolic profile. Similarly, Ogunade and McCoun (2020) measured LW on days 0 and 42 and then associated the metabolic profile of animals assessed at the end of the trial (i.e., blood collection on day 42). The latter authors found that metabolites including prolyl-valine, prolyl-iso-leucine, and prolyl-leucine were greater in steers with an improved growth rate. In the light of these results, those metabolites reported by Connolly et al. (2019) and Ogunade and McCoun (2020) may be associated with animals’ growth rate over the entire trial period whereas those correlations found in the present study could explain the shorter-term growth rate. However, these are only speculations based on Connolly et al. (2019) and Ogunade and McCoun (2020) because no LW data was collected at different intervals of time to calculate growth rate and support such hypotheses.

Our results reveal that associations with LWC were strongest in some metabolites whereas a lack of association was observed for other metabolites regardless of LWC calculations performed. Nevertheless, the main objective of the present study was not to discuss the metabolism behind metabolites’ appearance but variations in metabolites’ associations according to LWC calculations. Changes in the growth path of grazing cattle over time from the present study are fully discussed by Imaz et al. (2019) and its relationships with the animals’ metabolome across different feeding scenarios are discussed by Imaz et al.(2022). In this regard, Imaz et al. (2022) hypothesised about the use of metabolites, such as branch amino acids and lipids, by animals showing contrasting growth rates. Additionally, the present study shows that some metabolites, such as citrate, could even increase their strength of association as LWC calculations are done using longer intervals of time. Our experimental design does not enable us to conclude on these hypotheses and their relationship with different LWC calculations described in the present study but certainly stresses the importance of considering the time frame in which response variables are measured and biological samples are taken according to the group of metabolites found and mechanisms potentially involved in observed responses.

The outcomes of the present study could guide research that aims to analyse biological samples (e.g., blood, faecal samples) to understand biological mechanisms affecting traits of interest. For instance, weaners’ data presented indicate that LW measured 56 days from the metabolome assessment resulted in an estimated LWC of 0.114 kg/d; however, LWC1 was 0.507 kg/d being 4-fold greater. Results and conclusions could be very different depending on the periods of time LWC was represented. Therefore, investigations on associations between cattle growth rate and metabolites’ abundance measured from biological samples should consider measuring LWC at intervals shorter than 28 days. Additionally, future studies should determine whether the frequency of measurements on animals and the time when their metabolic profile is assessed could affect other traits which may show a different variation over time (e.g., slow or moderate changes in a particular trait over long periods of time). For instance, Novais et al. (2019) recorded feed efficiency in beef cattle for 70 days and studied associations with serum collected once at the beginning of a 21-day adaptation period before the feed efficiency evaluation. Similarly, Gómez et al. (2020) studied metabolic differences between cattle breeds during an embryo transfer program by collecting blood samples on day 0 (oestrus) and day 7 before an embryo transfer. Gómez et al. (2020) found that differences in metabolism amongst breeds were more evident on day 0 than on day 7. Studies should evaluate the impacts of the number and point in time to collect biological samples while phenotyping animals over the entire trial period. Additionally, results from Gómez et al. (2020) demonstrated that the metabolic profile of different breeds could differ even within a weekly period. These suggest that changes in animals’ metabolism that occur during short periods could not be detected if both measurements on animals and metabolome assessments are not performed at the right time and with enough frequency.

## Conclusions

Findings from the present study reveal that rapid changes in grazing cattle metabolome may not be reflected in associations with live weight change if they are not assessed close in time. Our results suggest that live weight change should be measured for a period shorter than 28 days before the metabolome assessment to better understand the metabolic pathways involved.

## Data Availability

The databases from the present study are available from the corresponding author on reasonable request.
